# Right ventricular adaptation in congenital heart disease: Does the type of right ventricular overload matter?

**DOI:** 10.1016/j.xjon.2025.08.013

**Published:** 2025-09-08

**Authors:** Renée S. Joosen, Gregor J. Krings, Heleen B. van der Zwaan, Nefise Karaman, Marco Guglielmo, Lucas R. Celant, Marco J.W. Götte, Michael G. Dickinson, Michiel Voskuil, Marielle C. van de Veerdonk, Johannes M.P.J. Breur

**Affiliations:** a Department of Pediatric Cardiology, University Medical Center Utrecht, Utrecht, The Netherlands; b Department of Cardiology, University Medical Center Utrecht, Utrecht, The Netherlands; c Department of Pulmonary Medicine, Amsterdam UMC Location Vrije Universiteit Amsterdam, The Netherlands; d Pulmonary Hypertension and Thrombosis, Amsterdam Cardiovascular Sciences, Amsterdam UMC Location Vrije Universiteit Amsterdam, The Netherlands; e Department of Cardiology, Amsterdam University Medical Centers, Amsterdam Cardiovascular Sciences, University of Amsterdam, Amsterdam, The Netherlands

**Keywords:** congenital heart defects, right ventricle, cardiac magnetic resonance, heart catheterization, hemodynamics

## Abstract

**Background:**

Right ventricular (RV) function is an independent predictor of prognosis in congenital heart disease (CHD), but RV adaptation to chronic pressure and/or volume overload is understudied. This study aimed to assess adaptation to chronic RV pressure and/or volume overload in CHD patients using pressure–volume (PV) loop analysis.

**Methods:**

This retrospective study included CHD patients with a subpulmonary morphologic right ventricle and biventricular circulation who underwent right heart catheterization (RHC) and cardiac magnetic resonance (CMR) imaging within 12 months prior to RHC at the University Medical Center Utrecht between August 2013 and November 2024. RV volumes, function, pressures and wall tension were obtained. RV afterload (arterial elastance [Ea]), contractility (end-systolic elastance [Ees]), RV–pulmonary artery (PA) coupling (Ees/Ea), and diastolic stiffness (end-diastolic elastance [Eed]) were evaluated using PV loop analysis.

**Results:**

Forty-five patients (67% male; median age, 14 [interquartile range, 9-17] years; 18 with pressure overload, 7 with volume overload, 20 with combined overload) were included. Pressure and combined overload led to a twofold increase in Ea, increased Ees, and increased Eed compared to volume overload (all *P* < .05), while volume and combined overload increased end-diastolic volumes (*P* = .010). RV-PA coupling, wall tension, and CMR-derived mass, function, and strain were similar across groups.

**Conclusions:**

Despite similar RV function on conventional imaging, PV analysis revealed distinct RV adaptation patterns for RV pressure and/or volume overload in CHD, suggesting potential for RV overload-specific treatments.

**Figure:**
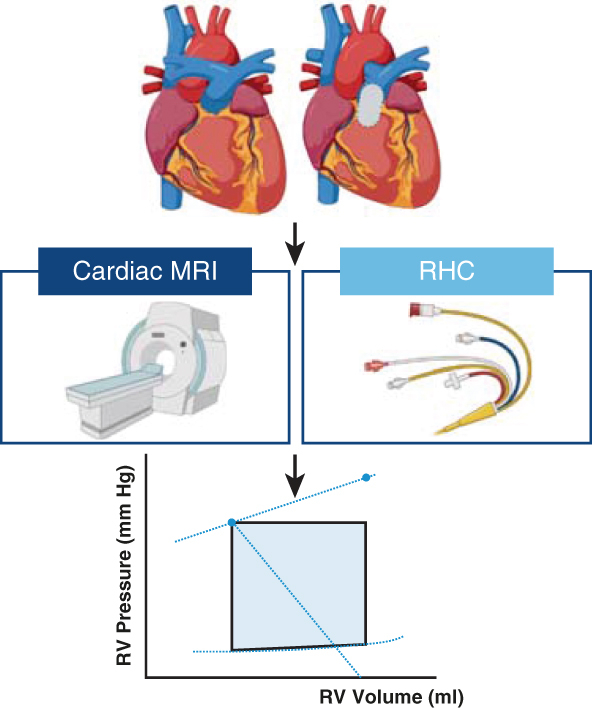
Single-beat pressure volume loop analysis was performed in patients with RV pressure and/or volume overload by combining RV pressures from right heart catheterization with RV volumes from cardiac MRI. Created in BioRender. Joosen, R. (2025) *MRI*, magnetic resonance imaging; *RHC*, right heart catheterization; *RV*, right ventricle.

**Central Message**

Despite similar RV function on conventional imaging, PV analysis revealed distinct RV adaptation patterns for RV pressure and/or volume overload in CHD.

**Perspective**

Differences in RV adaptation to various overload types in CHD offer the opportunity to develop targeted strategies. Tailored reintervention timing and therapies to boost RV contractility or manage volume could greatly improve outcomes. These findings also may shed light on RV adaptation in other conditions involving RV overload.

Right ventricular (RV) function is an independent predictor of prognosis in patients with congenital heart disease (CHD).^1^ It is influenced by RV pressure and/or volume overload, which may arise from the congenital abnormality itself or from residual lesions after surgical or percutaneous intervention. Long-standing pressure overload results in concentric RV hypertrophy, postsystolic flattening of the interventricular septum, and eventually progressive RV dilatation. Conversely, volume overload results in RV dilatation, eccentric RV hypertrophy and post-diastolic flattening of the interventricular septum. These RV adaptational mechanisms all serve to maintain cardiac output despite the altered loading conditions.^2^^,^^3^ Generally, RV volume overload is better tolerated than RV pressure overload, owing to the RV's more compliant, thinner wall^4^; however, prolonged states of RV overload can lead to RV maladaptation, resulting in RV dysfunction and failure, arrhythmias, decreased exercise capacity, and impaired quality of life.^5^, ^6^, ^7^ Therefore, early recognition of RV maladaptation is crucial to allow for timely interventions.

Current methods for assessing RV function, including RV ejection fraction (RVEF) and strain using cardiac magnetic resonance (CMR) imaging and transthoracic echocardiography, have limitations. They are highly load-dependent and focus on robust parameters (RVEF and volumetric parameters) that may fail to detect subclinical RV dysfunction, potentially missing the window when myocardial damage is still reversible.^8^^,^^9^ RV pressure–volume (PV) loop analysis overcomes these limitations and is considered the gold standard for evaluating RV adaptation in relationship to its load and. Therefore, it may predict maladaptive remodeling.^8^ However, the gold standard PV loop analysis using a specialized conductance catheter (multibeat method) is limited by technical challenges and the need for preload-altering maneuvers (eg, vena cava occlusion, liver compression, passive leg raises, volume challenges), making it unsuitable for some patients.^8^^,^^10^ To address these limitations, the single-beat method has been developed and validated as a reliable and practical alternative approach.^11^

To date, RV PV loop analysis has been conducted in only a small number of patients with CHD, and the impact of chronic RV pressure and/or volume overload on RV adaptation in CHD remains largely unknown.^9^^,^^12^^,^^13^ Therefore, this study aimed to assess the impact of chronic RV pressure and/or volume overload on RV adaptation patterns in CHD patients using right heart catheterization (RHC), CMR imaging, and PV loop analysis via the single-beat method.

## Methods

### Patient Population

All patients with CHD characterized by a subpulmonary morphologic RV in a biventricular circulation who underwent RHC for RV pressure and/or volume overload and a CMR within 12 months prior to the RHC at the University Medical Center Utrecht between August 2013 and November 2024 were identified retrospectively from our heart catheterization database. Patients were excluded if RV pressure curves or CMR images were unavailable. Patients were divided into 3 subgroups: (1) RV pressure overload, defined as an invasively measured RV systolic pressure >30 mm Hg at rest during RHC performed under continuous anesthesia^14^; (2) RV volume overload, defined as ≥20% pulmonary regurgitation or the presence of a shunt using CMR; and (3) both RV pressure and volume overload, or combined RV overload. This study was approved by the Institutional Ethics Committee of the University Medical Center Utrecht (study 24U-0036, approved July 9, 2024). Written informed consent for publication was obtained for all patients.

### RHC

RHC was conducted under continuous anesthesia. During the procedure, invasive pressure measurements were obtained from the right atrium, right ventricle, main PA (MPA), right PA, and left PA. PA compliance was calculated as the ratio of stroke volume (SV) to pulmonary pulse pressure (the difference between pulmonary systolic pressure and pulmonary diastolic pressure).^15^

### CMR

CMR images were obtained using a 1.5-T scanner (Philips Medical Systems) with the patient awake throughout the scan. Postprocessing was performed by an experienced observer (R.S.J.) using Circle Cardiovascular Imaging (CVI42; version 5.12.4) to acquire biventricular volumes, mass, and function. he feature-tracking module was used to obtain RV free wall global longitudinal strain (GLS), left ventricular GLS, and biventricular circumferential strain (CS). Endocardial and epicardial borders were manually drawn in a stack of short-axis images in the end-diastolic phase and the end-systolic phase. Papillary muscles and trabeculations were included in the ventricular wall mass. SV was calculated as end-diastolic volume (EDV) – end systolic volume (ESV). Ejection fraction was acquired using SV/EDV × 100. Biventricular wall mass was obtained by diastolic mass + systolic mass/2, to reduce observer variability.^16^ Biventricular relative wall thickness was calculated as mass/EDV.^17^ Right ventricular end-systolic wall tension was calculated according to the law of Laplace: 0.5 × RV systolic pressure × RV end-systolic radius divided by RV end-systolic wall thickness.^18^ Volumes and masses were indexed to body surface area according to the formula of Dubois and Dubois.

### PV Loop Analysis

Single-beat RV PV loop analysis was used to assess the interaction between the RV and PA load as described before.^9^ RV end-systolic elastance (Ees), a load-independent indicator of ventricular contractility, was calculated at rest using the following formula: RV maximal isovolumic pressure (RV Piso) − RV systolic pressure (RV Psys)/SV.^19^ RV maximal isovolumic pressure (Piso) was estimated using sine wave extrapolation, based on RV pressure values recorded just before the peak of the first derivative of pressure development (dP/dt) and just after the minimum dP/dt. Piso, the predicted maximal pressure during isovolumic contraction of the RV, is a validated method for determining RV maximal isovolumic pressure.^11^ To account for respiratory variations, RV pressure curves were averaged over multiple cardiac cycles. Pulmonary arterial elastance (Ea), reflecting mainly pulmonary vascular resistance (PVR), was calculated using the formula: RV Psys/SV.^20^ The ratio of Ees to Ea quantifies the efficiency of mechanical energy transfer from the RV to the pulmonary vasculature (RV-PA coupling), illustrating the RV's ability to adapt to varying loading conditions.^20^ RV end-diastolic elastance (Eed) was determined by fitting a nonlinear curve through the origin (0,0), as well as the begin-diastolic and end-diastolic points on the PV curve. Eed was calculated as the slope of this curve at end-diastolic volume, representing the diastolic stiffness of the right ventricle.^21^

### Statistical Analysis

Statistical analysis was conducted using SPSS version 29.0.1 (IBM). Figures were created with Prism version 10.2.0 (GraphPad Software). Categorical variables were expressed as frequency (percentage), with differences analyzed using the χ^2^ test or Fisher exact test. Continuous variables were tested for normality using a Q-Q plot. Non-normally distributed variables were presented as median (interquartile range [IQR]); normally distributed variables, as mean ± standard deviation. Correlations were evaluated using the Pearson correlation test for normally distributed data or the Spearman correlation test for non-normal data.

Differences among the RV overload groups were analyzed using one-way analysis of variance (ANOVA) with Bonferroni post hoc analysis for normally distributed continuous outcomes and the Kruskal-Wallis test with the Mann-Whitney *U* post hoc test for non-normally distributed variables. If the Levene test for homogeneity of variances was not met for ANOVA, a Welch ANOVA with Games-Howell post hoc analysis was used. Statistical significance was defined as a 2-tailed *P* value < .05.

## Results

### Patient Characteristics

Characteristics of the study cohort are summarized in Table 1. A total of 45 patients were included, with the majority being male (67%). Thirteen patients (29%) were diagnosed with transposition of the great arteries (TGA), almost all of whom (92%) had undergone a Lecompte procedure. Within the TGA group, 8 patients (62%) had an intact ventricular septum, 2 had a ventricular septal defect, and 2 were diagnosed with a double-outlet right ventricle with subpulmonary ventricular septal defect (Taussig-Bing anomaly). Nineteen patients (42%) had Tetralogy of Fallot (ToF), including 1 patient with a 22q11 deletion. Among the ToF group, 12 patients (63%) underwent repair with a transannular patch (TAP). The remaining 13 patients (29%) had other diagnoses, including pulmonary valve stenosis (n = 2), truncus arteriosus (n = 2), Ross procedure (n = 5), Ebstein anomaly with atrial septal defect type II (n = 1), and atrial septal defect type II (n = 3). The median age at the time of primary repair was 37 days (IQR, 7-162 days), and the median weight during the primary repair was 5.6 kg (IQR, 3.7-7.2 kg). Eighteen patients (40%) had RV pressure overload, 7 (16%) had RV volume overload, and 20 patients (44%) had combined RV overload. The median time interval between CMR and RHC was 4 months (IQR, 2-6 months).

**Table 1:** Patient characteristics (N = 45)

Characteristic	Value
Male sex, n (%)	30 (67)
Age, y, median [IQR]	14 [9-17]
Diagnosis, n (%)	
TGA	13 (29)
Simple TGA	8 (62)
Lecompte	12 (92)
ToF	19 (42)
22q11 deletion	1 (5)
TAP	12 (63)
Other	13 (29)
PV stenosis	2 (15)
Truncus arteriosus	2 (15)
Post–Ross procedure	5 (38)
Ebstein anomaly with ASD type II	1 (8)
ASD type II	3 (23)
Age at primary repair, d, median [IQR]	37 [7-162]
Weight primary repair, kg, median [IQR]	5.6 [3.7-7.2]
RV overload, n (%)	
Pressure	18 (40)
Volume	7 (16)
Pressure and volume	20 (44)
Time between CMR and RHC, mo, median [IQR]	4 [2-6]
Surgical or percutaneous reinterventions after RHC, n (%)	12 (27)

*IQR*, Interquartile range; *TGA*, transposition of the great arteries; *ToF*, tetralogy of Fallot; *TAP*, transannular patch; *PV*, pulmonary valve; *ASD*, atrial septal defect; *RV*, right ventricular; *CMR*, cardiac magnetic resonance; *RHC*, right heart catheterization.

### RV Pressure Versus Volume Overload

The RV pressure overload group and combined RV overload group exhibited nearly twice the RV systolic pressures compared to the RV volume overload group (median RV systolic pressure: 44 [IQR, 37-52] mm Hg; volume: 27 [IQR, 19-29] mm Hg; combined: 45 [IQR, 37-63] mm Hg; *P* < .001) (Table 2). Indications for RHC due to RV pressure overload included pulmonary valve stenosis (n = 12), MPA stenosis (n = 4), bilateral PA stenosis (n = 4), and unilateral PA stenosis (n = 18). The distribution of unilateral PA stenosis versus PV stenosis, MPA stenosis, or bilateral PA stenosis was similar in both the RV pressure overload group and the combined RV overload group. No increased invasive PA pressures were observed, and pulmonary vein drainage remained unaffected in all patients. In both volume groups and the combined group, RV volume overload was related mainly to severe pulmonary valve insufficiency (n = 23; mean value, 32 ± 10%) or the presence of a shunt (n = 4; mean Qp:Qs, 1.4 ± 0.4). Patients with RV volume overload and combined RV overload had a significantly larger mean RV end-diastolic volume (RVEDV) compared to those with only RV pressure overload (pressure: 95 ± 26 mL/m^2^; volume: 121 ± 16 mL/m^2^; combined: 117 ± 25 mL/m^2^; *P* = .010) (Table 3).

**Table 2:** Invasive heart catheterization and PV loop data

Parameter	Pressure (N = 18)	Volume (N = 7)	Combined (N = 20)	*P* value
Heart catheterization				
HR, bpm, mean ± SD	73 ± 13	67 ± 10	74 ± 14	.563
SBP, mm Hg, mean ± SD	82 ± 8	86 ± 13	80 ± 12	.637
RA Psys, mm Hg, median [IQR]	9 [7-10]	10 [9-11]	9 [7-10]	.464
RV Psys, mm Hg, median [IQR]	44 [37-52]∗	27 [19-29]∗^,^†	45 [37-63]†	**<.001**
RVEDP, mm Hg, median [IQR]	11 [7-13]	7 [4-10]	12 [7-13]	.052
LV Psys, mm Hg, mean ± SD	93 ± 7	83 ± 1	96 ± 13	.078
LVEDP, mm Hg, mean ± SD	13 ± 6	11 ± 3	11 ± 3	.868
PA Psys, mm Hg, median [IQR]	26 [24-35]	20 [17-28]	29 [21-34]	.178
mPAP, mm Hg, median [IQR]	18 [16-23]∗	12 [10-16]∗	16 [13-19]	**.022**
PAC, mL/mm Hg, median [IQR]	5 [3-6]	7 [4-9]	4 [3-6]	.217
PV loop analysis				
RV afterload (Ea), median [IQR]	0.60 [0.45-0.76]∗	0.27 [0.18-0.32]∗^,^†	0.73 [0.47-1.01]†	**<.001**
RV contractility (Ees), median [IQR]	0.35 [0.16-0.82]	0.14 [0.11-0.28]†	0.61 [0.34-1.01]†	**.032**
RV-PA coupling (Ees/Ea), median [IQR]	0.60 [0.35-1.04]	0.75 [0.44-1.6]	0.85 [0.52-1.18]	.504
RV diastolic stiffness (Eed), median [IQR]	0.33 [0.21-0.47]∗	0.10 [0.04-0.14]∗^,^†	0.33 [0.21-0.47]†	**.001**

*HR*, Heart rate; *SD*, standard deviation; *SBP*, systolic blood pressure; *RA*, right atrial; *Psys*, systolic pressure; *IQR*, interquartile range; *RV*, right ventricular; *RVEDP*, right ventricular end-diastolic pressure; *LV*, left ventricular; *LVEDP*, left ventricular end-diastolic pressure; *PA*, pulmonary artery; *mPAP*, mean pulmonary artery pressure; *PAC*, pulmonary artery compliance; *Ea*, arterial elastance; *Eed*, end-diastolic elastance; *Ees*, end-systolic elastance.

∗ *P* < .005 pressure versus volume.

† *P* < .005 volume versus combined.

**Table 3:** Cardiac magnetic resonance data

Parameter	Pressure (N = 18)	Volume (N = 7)	Combined (N = 20)	*P* value
RVEDV, mL/m^2^, mean ± SD	95 ± 26∗,†	121 ± 16∗	117 ± 25†	**.010**
RVESV, mL/m^2^, median [IQR]	40 [32-57]	69 [48-79]	63 [46-77]	**.043**
RVSV, mL/m^2^, mean ± SD	48 ± 8	58 ± 7	56 ± 12	**.025**
RVEF, %, mean ± SD	52 ± 10	49 ± 8	48 ± 8	.326
RV mass, g/m^2^, mean ± SD	24 ± 9†	23 ± 9‡	32 ± 7†,‡	**.007**
RV relative wall thickness, g/mL, mean ± SD	0.25 ± 0.07	0.18 ± 0.05‡	0.29 ± 0.08‡	**.013**
RV wall tension, mm Hg, median [IQR]	238 [189-274]	192 [157-219]	217 [178-318]	.287
RV FWGLS, %, mean ± SD	−21 ± 5	−20 ± 5	−19 ± 4	.550
RV GCS, %, median [IQR]	−15.5 [−17.5 to −13.5]	−14.0 [−14.6 to −10.3]	−17.0 [−17.9 to −13.2]	.135

Bold type indicates statistical significance, *RV*, Right ventricular; *EDV*, end-diastolic volume; *SD*, standard deviation; *ESV*, end-systolic volume; *IQR*, interquartile range; *SV*, stroke volume; *EF*, ejection fraction; *FWGLS*, free wall global longitudinal strain; *GCS*, global circumferential strain.

∗ *P* < .005 pressure versus volume.

† *P* < .005 pressure versus combined.

‡ *P* < .005 volume versus combined.

### Adaptation to RV Overload

**Figure 1:**
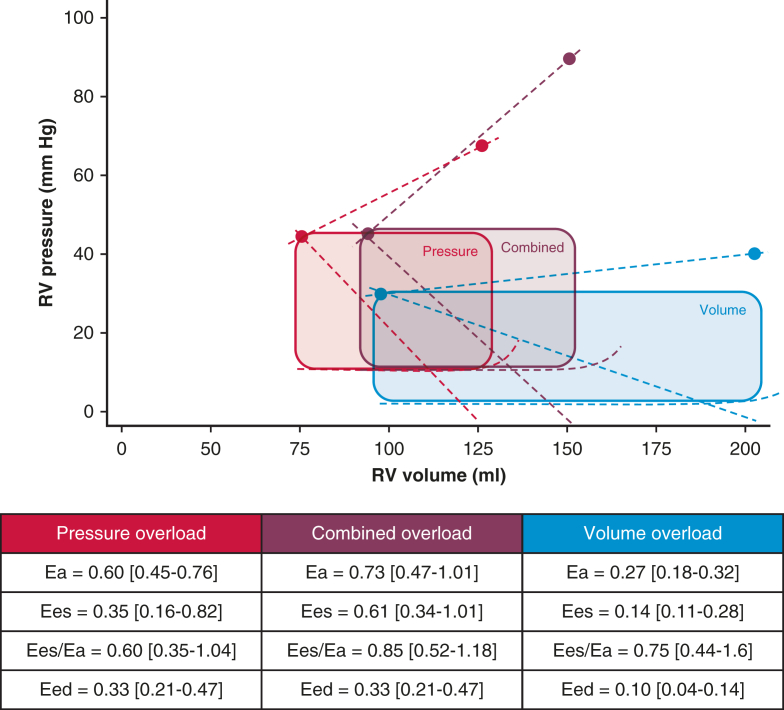
Pressure–volume (PV) loops in different groups of right ventricular (RV) overload. Schematic results of RV PV loop analysis in groups of RV pressure overload, RV volume overload, and RV pressure and volume overload (combined). Arterial elastance (Ea), mainly a reflection of pulmonary vascular resistance, was calculated as RV systolic pressure/SV.^20^ End-systolic elastance (Ees), a load-independent measure of ventricular contractility, was calculated as (RV maximal isovolumic pressure – RV systolic pressure)/SV.^19^ The maximal isovolumic pressure of the RV (Piso), used to analyze RV contractility (Ees), was computed by sine wave extrapolation using RV pressure values recorded before maximal first derivative of pressure development over time (dP/dt) and after minimal dP/dt.^19^ Right ventricular–-to-pulmonary arterial coupling was calculated as Ees/Ea and represents the efficiency of energy transfer from the RV to the pulmonary vasculature RV. RV end-diastolic elastance (Eed), used to assess diastolic stiffness, was calculated as the slope of the end-diastolic PV relationship at end-diastole.^21^

PV loop analysis revealed significantly higher RV afterload (Ea) in the RV pressure overload and combined RV overload groups compared to the RV volume overload group (pressure: 0.60 [IQR, 0.45-0.76] mm Hg/mL; volume: 0.27 [IQR, 0.18-0.32] mm Hg/mL; combined: 0.73 [IQR, 0.47-1.01] mm Hg/mL; *P* < .001) (Table 2, Figure 1). A trend toward higher RV relative wall thickness also was observed across these groups, though no actual hypertrophy was present (pressure: 0.25 ± 0.07 g/mL; volume: 0.18 ± 0.05 g/mL; combined: 0.29 ± 0.08 g/mL; *P* = .013). Nevertheless, RV systolic pressure correlated with RV relative wall thickness (r = 0.649; *P* < .001).

**Figure 2:**
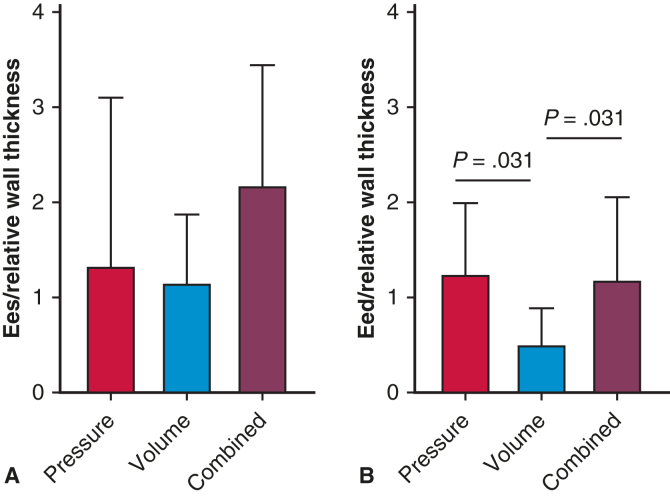
Right ventricular (RV) contractility and diastolic stiffness corrected for RV relative wall thickness. Graph shows RV wall thickness corrected end-systolic elastance (Ees) as measure of RV contractility among the 3 groups of RV overload (A) and RV wall thickness corrected end-diastolic elastance (Eed) as measure of RV diastolic stiffness in the 3 RV overload groups (B).

This pattern also was reflected by greater RV systolic pressures and RV relative wall thickness in patients with pulmonary valve stenosis, MPA stenosis, or bilateral PA stenosis compared to those with unilateral PA stenosis (RV pressure: 56 ± 14 mm Hg vs 39 ± 6 mm Hg [*P* < .001]; RV relative wall thickness: 0.32 ± 0.08 g/mL vs 0.22 ± 0.04 g/mL [*P* < .001]). RV contractility (Ees) was higher in the RV pressure and combined RV overload groups compared to the RV volume overload group, independent of RV relative wall thickness (pressure: 0.35 [IQR, 0.16-0.82] mm Hg/mL; volume: 0.14 [IQR, 0.11-0.28] mm Hg/mL; combined: 0.61 [IQR, 0.34-1.01] mm Hg/mL; *P* = .032) (Table 2, Figures 1 and 2). RV contractility (Ees) correlated with RV afterload (Ea) and RV systolic pressure (Ea: r = 0.517, *P* < .001; RV systolic pressure: r = 0.435, *P* = .003). RV pressure and combined RV overload groups demonstrated increased diastolic stiffness (Eed), independent of RV relative wall thickness (pressure: 0.33 [IQR, 0.21-0.47] mm Hg/mL; volume: 0.10 [IQR, 0.04-0.14] mm Hg/mL, combined: 0.33 [IQR, 0.21-0.47] mm Hg/mL; *P* = .001) (Table 2, Figures 1 and 2). RV Eed correlated with RVEDP (r = 0.600; *P* < .001), but not with RV relative wall thickness (r = 0.300, *P* = .050).

Despite variations in RV pressures, volumes, Ea, Ees, and Eed, similar RV-PA coupling (Ees/Ea) was observed across all groups (pressure: 0.60 [IQR, 0.35-1.04]; volume: 0.75 [IQR, 0.44-1.6]; combined: 0.85 [IQR, 0.52-1.18]; *P* = .504). Additionally, biventricular global function and strain were within normal limits, with no differences among the 3 groups (RVEF, 50 ± 8%; RV free wall GLS, −20 ± 5%; RV CS, −15 ± 3%) (Table 3; Table E1). RV wall tension was similar across the 3 groups (*P* = .287) and correlated with RVEF (r = −0.501; *P* < .001).

## Discussion

**Figure 3:**
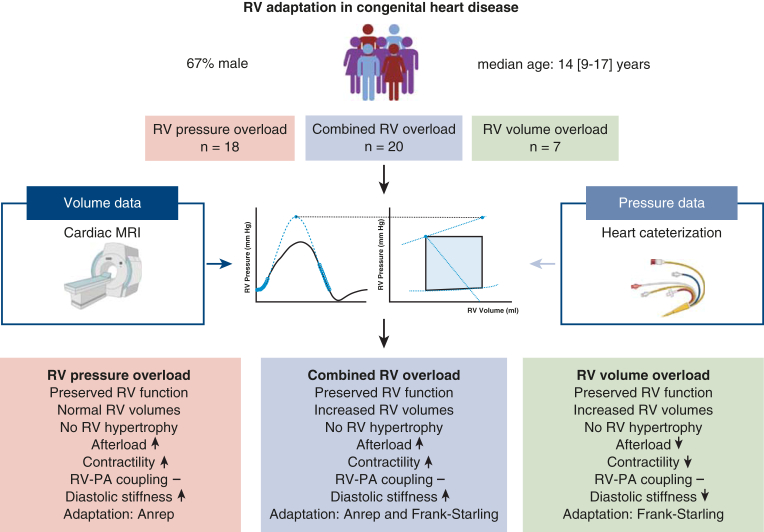
Right ventricular adaptation in congenital heart disease. Created in BioRender. Joosen, R. (2025) https://BioRender.com/p94z452. *PA*, Pulmonary artery; *RV*, right ventricle

This study is the first to evaluate the impact of chronic RV pressure and/or volume overload on RV adaption mechanisms in CHD patients using PV loop analysis. RV adaptation in chronic RV pressure overload is characterized by enhanced RV contractility, while chronic volume overload relies primarily on RV dilatation. In cases of combined pressure and volume overload, both mechanisms are seen, with RV pressure overload potentially mitigating the effects of volume overload. Despite distinct adaptation patterns among the RV overload subgroups, RV-PA coupling, RV wall stress, global RV function and strain were similar across the 3 groups (Figure 3).

### The Pressure-Overloaded Heart

We found that hearts with RV pressure overload, whether from pressure alone or from combined overload, showed increased RV pressure, Ea, and Ees values. There was a trend toward greater RV relative wall thickness and increased diastolic stiffness. Importantly, these changes occurred while RV global systolic function and strain were maintained. The preserved RV global systolic function and absence of severe RV hypertrophy in response to RV pressure overload align with previous studies in CHD patients^9^^,^^13^^,^^22^^,^^23^; however, to date few human studies have investigated the hemodynamic adaptation of the RV to overload, and none has compared different types of RV overload. In our previous work in TGA patients after the arterial switch operation and RV pressure overload due to PA stenosis, we described similar findings of increased afterload in the absence of RV hypertrophy, increased diastolic stiffness, and preserved RV systolic function.^9^

The results of the present study are also in line with several animal studies in which the pressure-overloaded RV was seen to adapt to increased afterload by enhanced myocardial contractility (the Anrep effect or homeometric autoregulation).^22^^,^^24^, ^25^, ^26^, ^27^, ^28^, ^29^, ^30^ Additionally, RV mass may increase as a compensatory response to elevated RV pressure to reduce wall stress, according to Laplace's law.^24^^,^^26^^,^^28^, ^29^, ^30^ However, despite a trend toward increased RV relative wall thickness, we found no increase in absolute RV wall mass. These findings are in contrast to the observations in adult patients with pulmonary arterial hypertension^16^ but are in line with previous studies in patients with pulmonary valve stenosis showing near-normal RV geometry, cavity size, low wall stress, and preserved global RV function.^23^ This raises the question of whether the RV has better adaptation mechanisms to pressure overload in patients with pulmonary valve stenosis compared to patients with acquired pulmonary arterial hypertension. Whether this might be explained by a more severe RV afterload in pulmonary arterial hypertension, a different response to RV pressure overload owing to retained fetal characteristics of the systemic RV, or both is unclear.^23^

In line with other studies, we observed increased RV diastolic stiffness, which may ultimately impair relaxation and filling, leading to reduced SV and increased RV preload reserve.^28^, ^29^, ^30^, ^31^ Although the underlying mechanisms in CHD remain unclear, several potential contributors to diastolic dysfunction have been proposed. RV hypertrophy might contribute to increased RV stiffening, leading to impaired filling; however, in the present study we found increased diastolic stiffness in the absence of RV hypertrophy. Moreover, we found significantly higher RV diastolic stiffness in the pressure and combined RV overload groups compared to the volume overload group, independent of RV relative wall thickness. These results suggest that additional intrinsic factors beyond RV wall thickness alone might contribute to increased diastolic stiffness. In line with our findings, various animal models of RV pressure overload have not demonstrated a relationship between RV hypertrophy and diastolic dysfunction.^32^ Other factors, such as increased collagen deposition and intrinsic stiffening of the RV cardiomyocyte sarcomeres, have been observed in other types of RV pressure overload, but any role in CHD remains to be investigated.^31^^,^^33^

### The Volume-Overloaded Heart

The RV volume overload group showed greater end-diastolic volume, lower afterload, lower contractility, and similar RV global systolic function, strain, and RV-PA coupling compared to the RV pressure overload and combined RV overload groups. This suggests that volume-overloaded RVs adapt primarily by increasing SV in response to elevated preload through the Frank-Starling mechanism (heterometric adaptation). The absence of the Anrep effect in isolated volume overload has been confirmed by human and animal studies.^13^^,^^24^^,^^25^^,^^27^^,^^28^ These RVs have been shown to have limited contractile reserves, and inotropic stimulation does not enhance their adaptation.^13^^,^^25^^,^^34^ Chronic volume overload leads to RV dilatation and disrupts the typical peristaltic contraction, potentially impairing the RV's ability to adapt through increased contractility.^27^ We also found low diastolic stiffness compared to the RV pressure overload group, which might facilitate adaptation via the Frank-Starling mechanism.^24^^,^^25^^,^^27^^,^^28^

### Combined (Pressure and Volume) Overload

Patients with combined RV overload exhibited features of both pressure and volume overload, including enhanced contractility and RV dilatation. These “phenotypes” might allow adaptation through both the Anrep effect and the Frank-Starling mechanism. Thus, interestingly, these patients might have a potential benefit. This benefit may lie in the combination of preserved contractility and impaired diastolic properties. A stiffer RV with maintained contractility appears better equipped to manage pulmonary regurgitation and resist progressive dilation over time.^25^^,^^28^^,^^35^ This is suggested by findings indicating that PA stenosis, as assessed by small branch PA diameter, is associated with a longer interval to pulmonary valve replacement.^36^

### Study Limitations

This study has several limitations. The small patient sample size in a heterogenous study population provides hypothesis-generating results; larger studies in more homogenous study populations are needed to confirm our findings and assess their clinical implications, including correlations with long-term outcomes. Comparisons were limited to between-group analyses due to the absence of an age- and sex-matched control group. Moreover, we were unable to differentiate the impact of volume overload type (shunt vs pulmonary regurgitation), which may influence RV adaptation and represents an important area for future research. Future research is also needed to establish normal values for RV adaptation to different types of RV overload in both children and adults with CHD. The time interval between measurements and the differing conditions under which they were performed (awake vs continuous general anesthesia) might have influenced our results. Therefore, our findings should be validated by directly comparing the single-beat and multibeat methods, using conductance catheters, in the CHD population. The lack of pulmonary arterial wedge pressure measurements hampered the calculation of pulmonary vascular resistance. Additionally, RV PV loop analysis was performed using the single-beat method. Although this approach relies on several assumptions, it serves as a reliable and practical alternative to the multibeat method, which is often infeasible in our patients due to technical challenges and the need for preload-altering maneuvers.^11^

### Clinical Implications

This study assessing the impact of RV overload on RV adaptive mechanisms in CHD patients using PV loop analysis reveals distinct patterns of RV adaptation to different types of RV overload. Understanding the differences in RV adaptation across the various types of RV overload in CHD patients will facilitate recognition of signs of RV maladaptation and failure within specific RV overload subgroups in clinical practice. Such insights have the potential to develop targeted RV overload-specific strategies aimed at optimizing the timing of reinterventions or reoperations, thereby reducing the burden of lifelong procedures. They also might lead to personalized solutions to support RV function, such as pharmaceutical therapies to improve RV contractility or fluid management, ultimately significantly enhancing patient outcomes. Over time, these findings are expected to contribute to clinical guidelines for managing RV failure and guiding decisions regarding reinterventions and reoperations in CHD patients. These findings also may help to better understand the tipping point at which RV adaptation shifts toward maladaptation in other groups of patients with chronic RV overload, such as those with RV pressure overload due to pulmonary hypertension or RV volume overload caused by tricuspid regurgitation or systemic-to-pulmonary shunts.

## Conclusions

Despite similar RV function as assessed on conventional imaging, PV analysis revealed distinct RV adaptation patterns for RV pressure and/or volume overload in CHD, suggesting the potential for RV overload-specific treatments.

## Conflict of Interest Statement

The authors reported no conflicts of interest.

The *Journal* policy requires editors and reviewers to disclose conflicts of interest and to decline handling or reviewing manuscripts for which they may have a conflict of interest. The editors and reviewers of this article have no conflicts of interest.

## Figures and Tables

**Table E1:** Cardiac magnetic resonance data, left ventricle

Parameter	Pressure (N = 18)	Volume (N = 7)	Combined (N = 20)	*P* value
LVEDV, mL/m2, mean ± SD	88 ± 16∗,†	71 ± 9∗	72 ± 14†	.002
LVESV, mL/m2, mean ± SD	37 ± 11∗,†	29 ± 4∗	27 ± 5†	.002
LVSV, mL/m2, mean ± SD	51 ± 9	42 ± 8	44 ± 9	.040
Cardiac output, L/min, mean ± SD	4.9 ± 1.6	6.9 ± 1.6	5.8 ± 2.3	.077
LVEF, %, mean ± SD	58 ± 7‡	59 ± 6	62 ± 5‡	.166
LV mass, g/m2, mean ± SD	58 ± 16	57 ± 11	56 ± 13	.896
LV relative wall thickness, g/mL, median [IQR]	0.68 [0.56-0.77]†	0.74 [0.66-1.03]	0.80 [0.67-0.86]†	.039
LV GLS, %, median [IQR]	−16.4 [−19.3 to −14.3]	−16.4 [−19.8 to −16.1]	−17.0 [−18.4 to −14.4]	.776
LV GCS (%), median [IQR]	−17.3 [−18.4 to −15.4]	−16.6 [−18.2 to −16.0]	−18.8 [−20.4 to −15.8]	.162

Bold type indicates statistical significance. *LV*, Left ventricular; *EDV*, end-diastolic volume; *SD*, standard deviation; *ESV*, end-systolic volume; *SV*, stroke volume; *EF*, ejection fraction; *IQR*, interquartile range; *GLS*, global longitudinal strain; *GCS*, global circumferential strain.

∗ *P* < .005 pressure versus volume.

† *P* < .005 pressure versus combined.

‡ *P* < .005 volume versus combined.
